# Cancer Cells Treated by Clusters of Copper Oxide Doped Calcium Silicate

**DOI:** 10.15171/apb.2019.013

**Published:** 2019-02-21

**Authors:** Mostafa Mabrouk, Sayed Hamed Kenawy, Gehan El-Tabie El-bassyouni, Ahmed Abd El-Fattah Ibrahim Soliman, Esmat Mahmoud Aly Hamzawy

**Affiliations:** ^1^Refractories, Ceramics and Building Materials Department, National Research Centre (NRC), 33 El Behooth St. Dokki-Giza, Egypt.; ^2^Pharmacognosy Department, Pharmaceutical and Drug Industries Division, National Research Centre (NRC), 33 El Behooth St. Dokki, Giza, Egypt.; ^3^Glass Research Department, National Research Centre (NRC), 33 El Behooth St. Dokki- Giza, Egypt.

**Keywords:** Copper oxide, Cytotoxicity, Doxycycline hyclate, Chemical precipitation

## Abstract

*** Purpose:*** Different compositions of copper oxide (CuO)-doped calcium silicate clusters were used to treat the cancer cells.

***Methods:*** The influence of CuO content on the morphology, drug delivering ability, physicochemical properties and cytotoxicity was investigated.

***Results:*** The microcrystalline structure revealed the decrement of the size from (20-36 nm) to (5-7 nm) depending on the copper content percentages. Drug delivering ability of doxycycline hyclate (Dox) was down regulated from 58% to 28%in the presence of the CuO. The inclusion of CuO and Dox didn’t show any remarkable changes on the physicochemical properties of the CuO-doped calcium silicate nanoparticles.

***Conclusion:*** The CuO-doped calcium silicate sample (5 weight %) exhibited great cytotoxicity against the tested cell lines compared to the CuO-free sample. CuO-doped materials displayed significant anticancer effect; this sheds light on its implication in the treatment of cancer.

## Introduction

One of the major challenges that face researchers targeting the cancer treatment was the direct delivering of the lethal dose only to cancer cells without killing the normal cells. To meet this challenge, biomedical researchers were looking for reliable local anticancer implants. For the treatment of cancer, the diseased tissue was surgically removed, resulting in a tissue defect, which was then remedied with tissue graft material.^1^ Considering material design, many researchers reported the implementation of calcium silicates for regenerative medicine.^2^

Preparation method had important role in controlling the properties of nanoparticles, including the particle size, morphology and chemical composition. These properties believed to be well controlled by controlling the wet precipitation method parameters such as precursor’s ratios, reaction temperature, additives sequence and the reaction time. All these parameters could be easily adjusted in the wet precipitation method in order to tolerate the aforementioned nanoparticles properties as confirmed by previous studies.^3,4^ In addition to that, low price precursors were used in the wet precipitation method compared to the sol-gel one.

Furthermore, addition of transition metals including copper oxide (CuO) ions to the biomaterial had altered a series of structural, physical, chemical, mechanical and biological properties, such as, solubility, resorption and bone bonding capability.^5,6^ Therefore, our research focused on the addition of various CuO concentrations to the calcium silicate during the preparation process using the wet precipitation method (economical and flexible method). CuO doping was expected to affect the reaction and the final properties of the resulting materials. Under this perspective, doping of calcium silicate with copper would promote bone cancer treatment and induce the bone regeneration.

## Materials and Methods

### Materials

The used materials were calcium carbonate (CaCO_3,_ 99%) from El-Gomhouria Company for Trading Chemicals and Medical Appliances (Egypt, Cairo), silica gel (SiO_2_, Fluka, Fluka Chemie GmbH, Parent company Sigma-Aldrich, Buchs, Switzerland] and copper carbonate [CuCO_3_, Fluka]. Analytical nitric acid (HNO_3_ 69%) and ammonia solution (25%) were delivered from Al-Ahram, Technical Chemical Laboratories, Giza, Egypt. As regard to the phosphate buffer the following ingredients were utilized KH_2_PO_4_,NaCl and KH_2_PO_4_ from Bio Basic Company (Markham, Canada).

### Synthesis of CuO-doped calcium silicate

**Figure 1:**
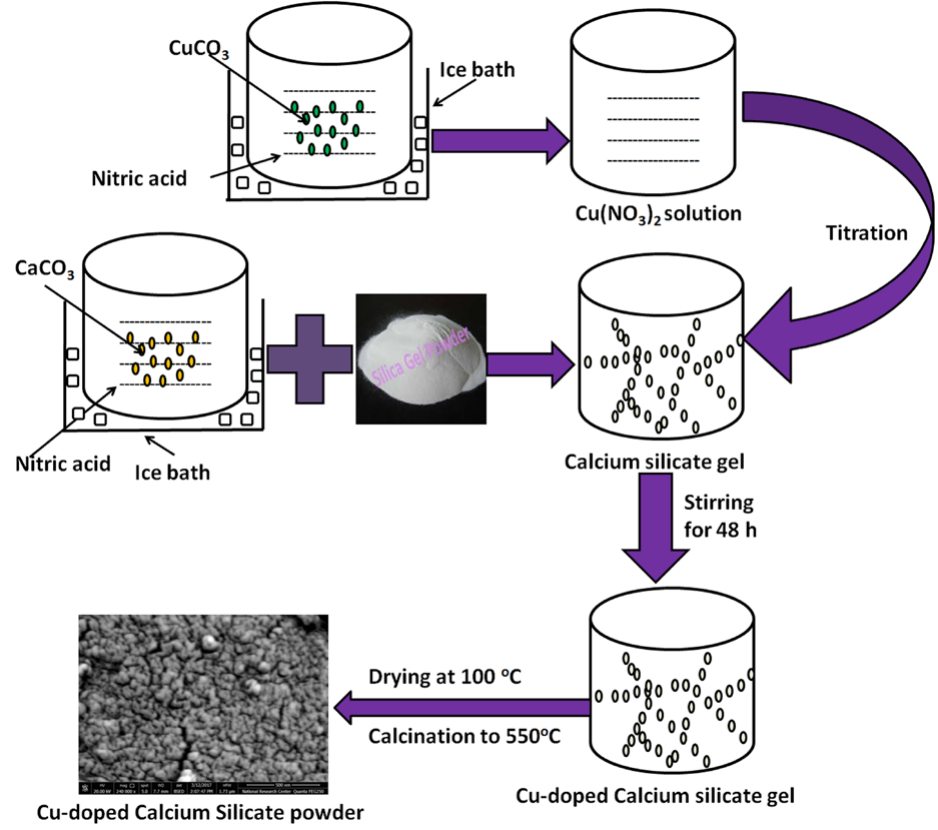
Schematic diagram for the wet precipitation method of CuO-doped calcium silicate (CuO-doped calcium silicate = copper oxide doped calcium silicate).

The compositions of CuO doped calcium silicate clusters were synthesized as illustrated in the schematic diagram Figure 1. CaCO_3_ (99%), SiO_2_ and copper carbonate (CuOCO_3_) were engaged as precursors for the Ca^2+^, SiO_2_ and CuO^2+^ respectively. Analytical nitric acid (HNO_3_) (mol : mol to the calcium carbonate) was used to ensure the reaction of all the used quantity of calcium carbonate with nitric acid to form calcium nitrate solution along with drop wise addition of ammonia solution (25%) to obtain gel like structure. In the present study, five individually weight % concentrations of the CuO^2+^ (1, 3, 5, 7 & 10) over/100 g of the CaSiO_3_ slurry was produced, the preparation route was mentioned elsewhere.^7^

### Effect of copper addition on the morphology

#### TEM analysis

Particle size of the CuO-doped nanoparticles was analyzed using transmission electron microscopy (TEM) [JEOL, Japan, JEM2100, Transmission Electron Microscope High-Resolution (TEM-HR)]. A trace amount of the nanoparticles about 10 mg was dispersed in distilled water, and few microliters (µL) were dropped onto a copper grid and TEM images were obtained.

#### SEM imaging

The morphology of the prepared CuO-doped nanoparticles was analysed using scanning electron microscopy (SEM) (JEOL JXA-840A, Electron Probe Micro-Analyzer, Japan) at 15 kV. The nanoparticles were coated with gold to improve the imaging of samples, then examined via SEM apparatus.

#### Drug loading and release

Doxycycline hyclate (Dox , 98% M. wt =512.94 g/mol, Sigma-Aldrich, Germany) was selected as an antibiotic model to treat the secondary bacterial infection that might occur during the implantation operation, due to its chemotherapeutic effects.^8,9^ Likewise, Dox has an ionic feature in the aqueous solution which causes the formation of an ion-complex with the CuO-doped nanoparticles as a multivalent counter-ion. Herein, an ion-complexed doxycycline was formed through blending of the CuO-doped powder in an aqueous solution of polyvinyl alcohol [3% PVA (^w^/_v_)] containing 15% Dox (^w^/_w_). The actual loaded Dox in the prepared samples was determined using the following equations.

(1) $$\text{\%Dox loading}=\frac{\text{Weight of Dox in formulation}}{\text{Weight of total formulation}}\times 100$$

(2) $$\text{\%Encapsulation Efficiency}=\frac{\text{Actual Dox loading}}{\text{Theoretical Dox loading}}\times 100$$

Moreover, the *in vitro* Dox release was measured in phosphate buffer solution (PBS [pH 7.4; 37°C]), after different time intervals, approximately 3 mL of the release medium was withdrawn and the concentration of Dox was spectrophotometrically determined at wavelength 273 nm using UV-Vis spectroscopy (Lambda 25 UV/Vis Spectrophotometer, PerkinElmer, Waltham, MA, USA) as early reported.^10^ Different mathematical models were applied to study the kinetics of the Dox-loaded systems.

#### The effect of copper addition on the physicochemical properties

In order to study the effect of CuO on the physicochemical properties of the prepared CuO-doped nanoparticles, infrared spectra were obtained using Fourier transformer infrared spectrophotometer (FT-IR) (model FT/IR-6100 type A, Germany). The spectra were recorded at wavenumbers range of 400–4000 cm^-1^. The measured samples were prepared by mixing nanoparticles with potassium bromide (KBr). Moreover, X-ray diffraction (XRD) analysis was used to comprehend the effect of CuO on the obtained phase of CuO-doped calcium silicate. The samples were analysed using XRD, BRUKER, D8 ADVANCED CuO target, Germany. Measuring rate of 2°/min and diffraction angle range of 2°-90°.

#### The cytotoxicity influence against cancer cell lines versus normal cells

The cell viability assay was conducted on the following cell lines, HepG2 (hepatocellular carcinoma), MCF-7 (breast adenocarcinoma) and HFB4 (normal melanocyte). The cell lines were purchased from ATCC (American Type Culture Collection). Practically, after 24 hours of seeding, the investigated cell lines were cultured in 96 well plates, the medium was changed to serum-free medium containing final concentration of the CuO-doped powder (100 μg/mL) in triplicates. Afterwards, the cells were incubated at the above mentioned conditions for 48 hours, doxorubicin (100 μg/mL) was used as positive control and 0.5% dimethyl sulfoxide (DMSO) was used as a negative control. Cytotoxicity and cell viability were determined using the MTT (3-(4, 5-dimethylthiazol-2-yl)-2, 5-diphenyltetrazolium bromide) assay.^11-13^

## Results and Discussion

### The effect of CuO on the morphology

**Figure 2:**
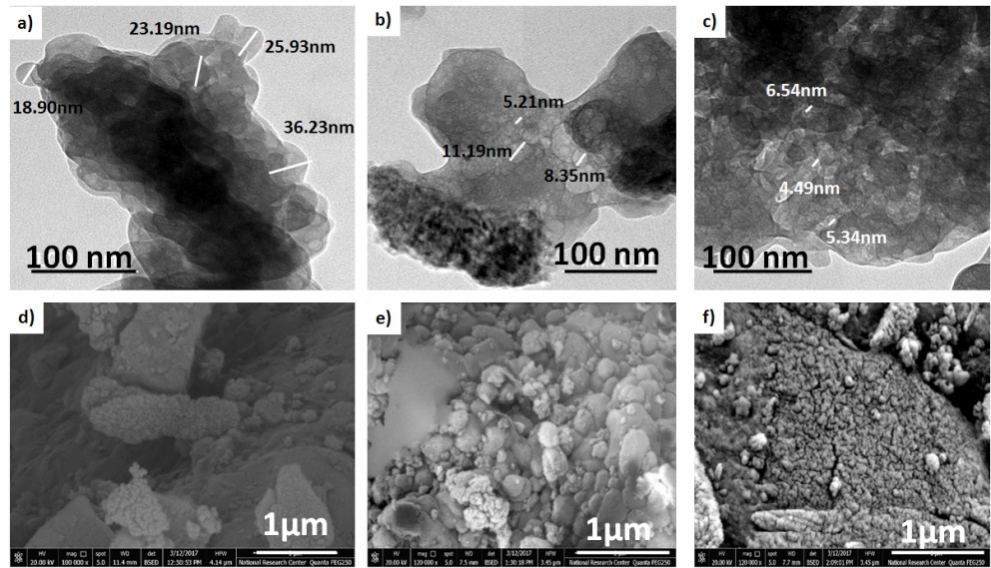
TEM image of (**a)** pure calcium silicate, (**b)** 3 % CuO-doped calcium silicate and (**c)** 5% CuO-doped calcium silicate and SEM images of **d)** pure calcium silicate, (**e)** 3 % CuO-doped calcium silicate and (**f)** 5% CuO-doped calcium.

Figure 2 represents the TEM images and the SEM micrographs. They displayed rounded to sub rounded grains in the nano-scale size ˂50 nm which ensure the nano-sized particles. Furthermore, the particle size of the prepared materials was found to be dependent on the CuO concentration. The higher the CuO content (5% CuO) the smaller the particle size was obtained (4.49-6.54 nm) compared to the CuO free sample (19.90-36.23 nm). It was worthy noted that the CuO-free sample revealed irregular agglomerated particles, while, the CuO-doped samples showed backed fine particles.

TEM= Transmission electron microscopy; CuO-doped calcium silicate = copper oxide doped calcium silicate.

### The drug delivering ability of Dox drug

#### In vitro drug release

**Figure 3:**
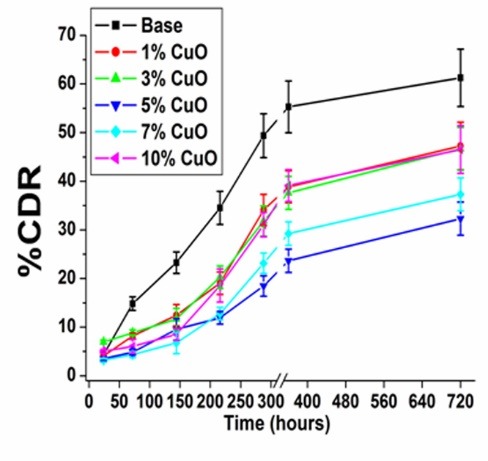
Dox release behaviour after immersion PBS for different time intervals (Dox = Doxycycline hyclate).

The entrapment efficiency values ranged from (96.38±0.70)% to (71.43±0.82)% with the increment of the CuO content. It is worth highlighting that the Dox release behaviour was affected with the CuO concentration. This result could be correlated to the ionic effect of the Dox with the released ions from the CuO-doped powder such as (CuO, Ca and Si).^14^ The percent of cumulative drug release (%CDR) profiles of the Dox-loaded samples were represented in Figure 3. The Dox release behaviour was down regulated from 58% to 28% in the presence of CuO. However, the release rate decrement was higher for the 5% CuO when compared to the samples containing higher CuO concentrations 7% and 10% respectively. These results elucidated that the CuO effect on the release behaviour was obtained at lower concentrations (up to 5%), while the higher CuO concentration would induce uncontrolled effect on the release behaviour, the same results were reported for the ciprofloxacin release behaviour from PAA-PCL scaffolds.^15^

#### Weight loss (%) and pH analysis

**Figure 4:**
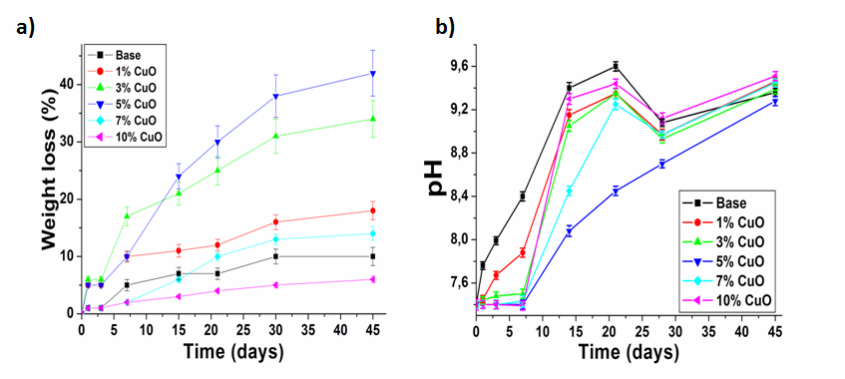
Illustrates (**a)** weight loss of the medicated samples and (**b)** pH value variations in the PBS. PBS, phosphate buffer saline.

The weight loss (%) of the Dox-loaded samples versus time was measured as presented in Figure 4a. The CuO-free sample showed 10 %weight loss. While, weight loss % in the 5% CuO-doped samples increased to about 40% after 45 days. This weight loss % was attributed to the PVA erosion and the powder dissolution in the PBS. However, in the samples with higher % of CuO concentration, the weight loss % was reversed. This result was in consistence with the *in vitro* drug release results. Evidently, controlling the CuO concentration in the fabricated nanoparticles may assist the samples degradation in the PBS.

Figure 4b showed the pH changes versus time of the PBS solution. The CuO-free and 1% CuO-doped samples elevated the pH value from 7.40 to 8.39 and 7.88 respectively during the first week. This could be attributed to the leakage of the calcium (Ca) and silicon (Si) ions from the samples into the PBS, while other CuO-doped samples did not demonstrate any significant increase in the pH value during the first week. This result was due to that the higher CuO concentration had retained the leakage of Ca and Si ions into the PBS. Subsequently, the pH values for all samples improved during the remaining immersion time due to the outflow of the Ca, Si and CuO into the PBS. The pH increment was found to be dependent on the CuO concentration. Previous study highlighted the relation between the weight loss (%) and the pH of the physiological media.^16^ The correlation between ions leakage and the sample degradation confirmed the ion-complexation theory for the Dox antibiotic as mentioned in the methodology section.

#### Dox release kinetics

The Dox kinetics release for the Dox-loaded samples was fitted using different mathematical models and the kinetic parameters were recorded in Table 1. The mathematical calculations revealed that the release of Dox from the CuO-free and 1% CuO followed the Ritger-Peppas models for non- swelling matrix. The release exponent (n) is 0.5 < n < 1.0 for the non-Fickian release (anomalous). This emphasized that the drug release followed both the diffusion and erosion controlled mechanisms. Furthermore, the CuO-loaded samples above the 1% followed the diffusion model as confirmed by theR^2^ values; in which the drug transferred from the solid surface into the PBS. These results confirmed our idea that the release behaviour and its kinetics were controlled by the leaked ions concentrations from the Dox-loaded samples (Ca, Si and CuO) into the PBS.^14,17^

#### The effect of CuO on the physicochemical properties

**Figure 5:**
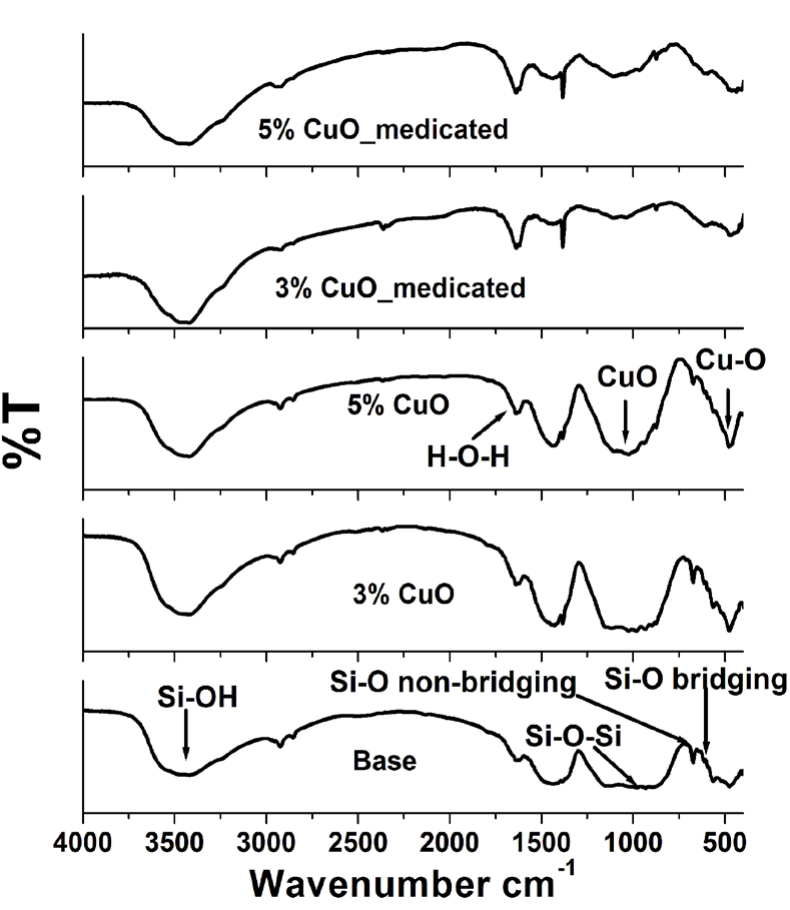
The FTIR spectrum of the medicated and non-medicated CuO-doped calcium silicate nano-powder. FTI R= Fourier-transform infrared spectroscopy; CuO-doped calcium silicate = copper oxide doped calcium silicate).

The chemical integrity of the prepared calcium silicate nanoparticles upon the addition of different concentrations of CuO and Dox antibiotic was investigated using FTIR. The FTIR spectra of selected different CuO-doped calcium silicate (0, 3 and 5) % respectively were compared to the same samples after medication as illustrated in Figure 5. Calcium silicate nanoparticles exhibited 6 infrared bands positioned at 613, 673, 892, 933, 981, and 1147 cm^-1^.^18^ Whereas those doped with 3% and 5% CuO showed approximately the same bands with minor shifts of the band 1147 cm^-1^ and 1105 cm^-1^ for 3 and 5 % samples, respectively. The bands at 613, 673, 892, 933, 981, and 1147 cm^-1^ were related to the silicate network and respectively ascribed to the Si-O symmetric stretching of bridging oxygen atoms between tetrahedrons, Si-O stretching of non-bridging oxygen atoms, Si-O-Si symmetric stretching, and the longitudinal-optical (LO) mode of the Si-O-Si asymmetric stretching.^19^ A weak band at 1609 cm^-1^ agrees to the asymmetric stretching vibration of the C-O bond and the broad band at 3254 cm^-1^ corresponded to the stretching mode in the hydroxide group.^20^

Moreover, the bands between the 2130-3419 and the 3468 cm^-1^ may possibly be due to the Si-OH stretching in Si-OH groups or to the stretching vibrations of the O-H groups in H_2_O or to the hydroxyls with a wide range of hydrogen bond strengths. The band at 1637 cm^-1^, was caused by the H-O-H bending vibration of molecular H_2_O.^21,22^ The spectrum of the 3 % CuO sample exhibited three vibrations at wave numbers 474, 563, and 593 cm^–1^, which might be familiar to the vibrations of CuO. While samples doped with 5% CuO showed bands at 476, 559, and 592 cm^-1^, confirming the formation of highly pure CuO NPs as showed in Figure 5. Both samples displayed a band at around 1025 cm^-1^ which may be ascribed to the CuO phase. Such assignments were in agreement with the available values in the literature.^23^ Moreover, it was observed that the CuO-doped characteristic bands around 400-1240 cm^-1^ diminished upon medication with Dox drug. These results suggested both expectations of complete inclusion of the nanoparticles within the PVA matrix and/or complexation of the nanoparticles with the Dox drug. The complexation between the Dox and CuO-doped calcium silicate was also ascertained by the increased intensities of the bands at 1638 and 3470 cm^-1^ assigned to H-O-H and Si-OH, respectively.

**Figure 6:**
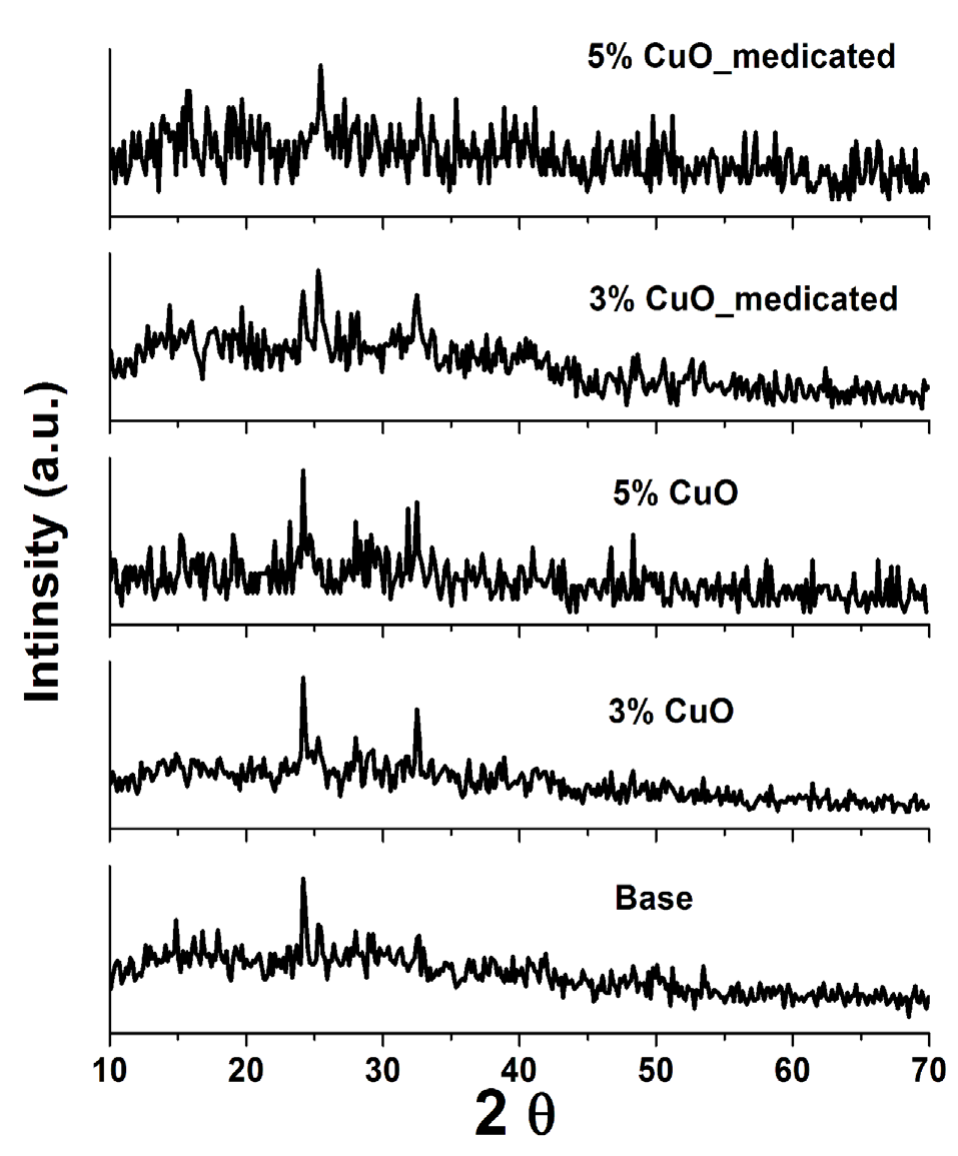
The XRD patterns of the medicated and non-medicated CuO-doped calcium silicate nano-powder. XRD= X-ray diffraction; CuO-doped calcium silicate = copper oxide doped calcium silicate.

In addition to the effect of CuO and Dox addition on the obtained phase of calcium silicate nanoparticles was investigated by XRD as shown in Figure 6. Semi-amorphous phases with small crystalline peaks were observed in all the prepared samples as a result of the low temperature used in the preparation of samples as previously pointed out in the processing section. The non-medicated nanoparticles demonstrated the semi-amorphous phase of quartz (82-1574 Card № SiO_2_), wollastonite (76-1846 Card № CaSiO_3_) and Ca-olivine (80-942 Card № Ca_2_SiO_4_). The addition of 5% CuO exhibited remarkable changes due to the appearance of the new [tenorite, CuO] phase as well as wollastonite and Ca-olivine phases. This result may be due to increasing the CuO amount that would in turn cause the formation of tenorite phase which has shown excellent matches with the standard ICDD Card № 00-005-0611coinciding with the previously reported study.^24^ The medicated patterns showed no remarkable changes compared to the non-medicated formulations. This finding suggested that the presence of Dox drug has no effect on the physical stability of the CuO-doped calcium silicate.

The obtained data of the microstructure and physicochemical properties of the prepared CuO-doped calcium silicate suggested that those properties are greatly influenced by the CuO concentration. This could be owed to the molar ratio between added silicate and metallic precursor related to the size and morphology of the synthesized silicate based materials as reported in previous studies.^25^ In the current research the molar ratio of Si and metallic precursors was changing with the CuO concentration while, the valence states of Ca and Cu ions were the same. Therefore, the molar quantity of positively charged ions possessed significant difference, which may account for the different ratios between Si and metallic precursors. Thus improves the microstructure and physicochemical properties of the prepared CuO-doped calcium silicate.

#### The cytotoxicity effect against cancer cell lines versus normal cells

**Figure 7:**
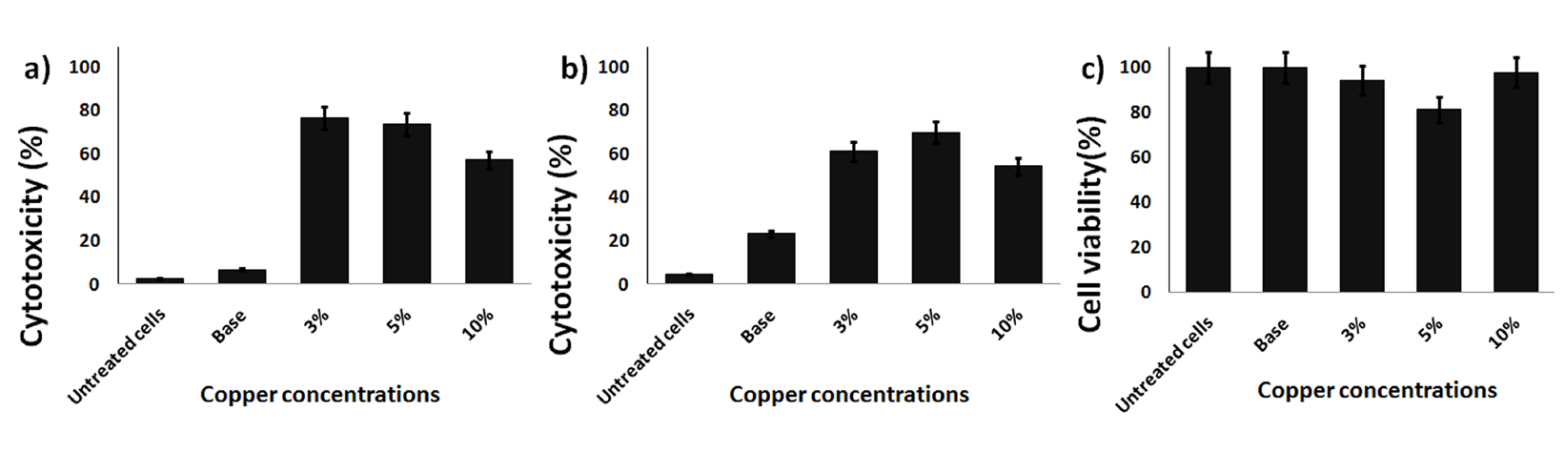
Cytotoxicity (%) of deferent concentrations of CuO-doped calcium silicate against (a) Hepatocellular carcinoma cell line (HepG2), (b) Breast cancer cell line (MCF-7) and (c) cell viability (%) of Normal Melanocyte (HFB4).

**Figure 8:**
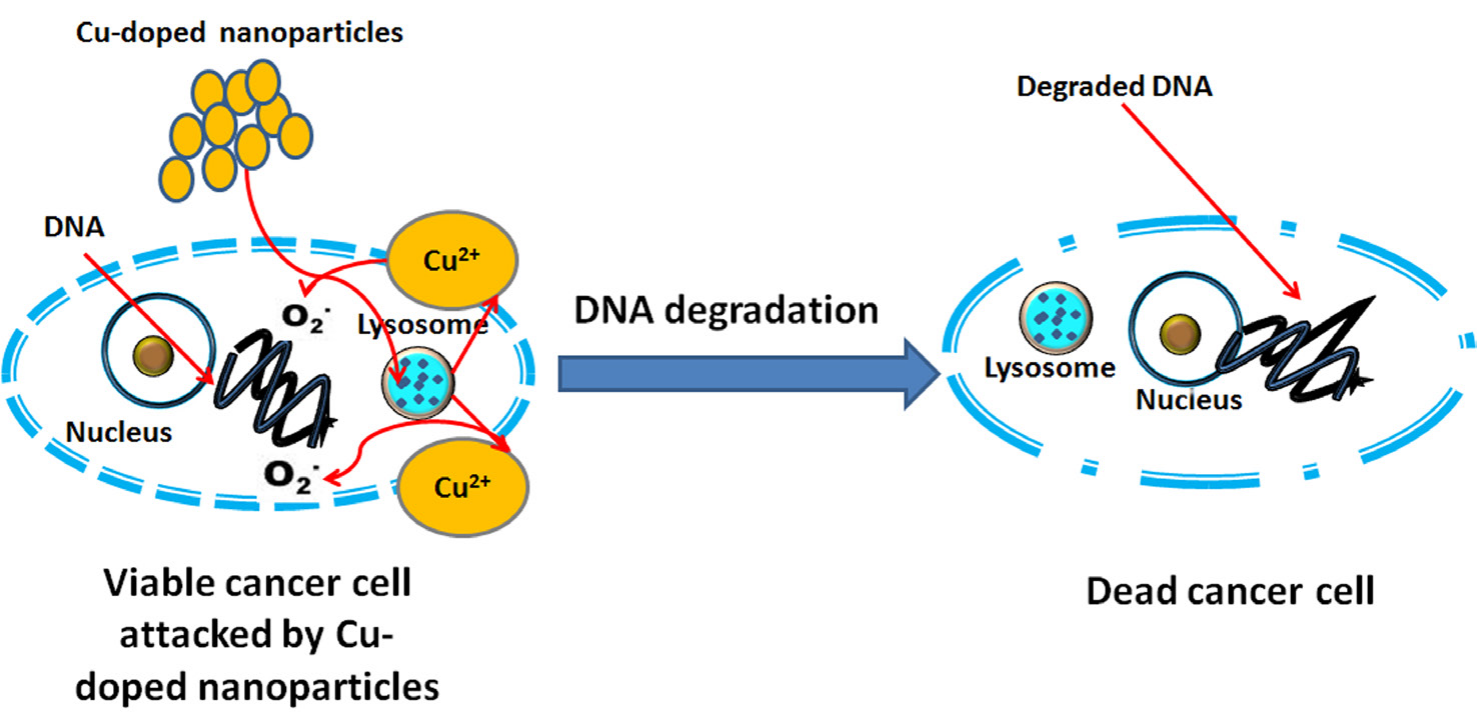
Suggested mechanism for the CuO anticancer activity. CuO, copper oxide.

The cytotoxicity consequences are shown in Figure 7. The results revealed that all the CuO-doped samples possessed high cytotoxic effect against the different cancer cell lines. On the other hand, the copper free sample exhibited a weak cytotoxic effect against all tested cancer cells. It is worth to highlight that (CuO) has a known effect on the cancer cells. Precisely, copper showed a dose-dependent degradation of DNA molecules through generation of oxygen.^26^ Therefore, copper nanoparticles are considered as an excellent candidate for targeted therapy. However, very minor effect for CuO was observed on the normal cells (HFB4) above 81.30% [Figure 7c). Thus the anticancer selectivity of the fabricated CuO-doped nanoparticles was delineated. The suggested mechanism of the CuO anticancer activity based on lysosomal degradation of CuO-doped nanoparticles was illustrated in Figure 8.

However, it is essential to highlight the role of copper in cancer pathogenesis and etiology. In our study, CuO-doped materials exhibited a higher DNA formation diminishing the killing of different cancer cell lines compared to the cisplatin complexes (a well-known chemotherapeutic drug).^27,28^ Several scientists’ have recently developed novel anticancer materials that involve dual CuO cores, in order to interact specifically with the two adjacent DNA phosphates. These phosphates are responsible for providing an active site for metallo-enzymes such as nucleases.^29,30^ The obtained results are promising and for full picture analysis of this system *in vivo* studies are required in the upcoming work.

**Table 1:** Release kinetics parameters of different Dox-loaded samples

**Formula code**	**R** ^ 2 ^ **-value**			**n**	**K**	**RE**_0-720h_ **(%)**
	**Zero-order**	**Diffusion**	**Korsmeyer–Peppas**			
Base	0.789	0.919	0.956	0.824	0.383	61.252
1%CuO	0.859	0.923	0.960	0.786	0.308	47.244
3%CuO	0.883	0.923	0.902	0.634	0.722	46.670
5%CuO	0.943	0.960	0.957	0.710	0.301	32.302
7%CuO	0.893	0.916	0.908	0.819	0.175	37.314
10%CuO	0.856	0.897	0.874	0.765	0.316	46.499

Note: n is the diffusion exponent; K is the release rate constant; RE _0-720h_(%) is the release efficiency of drug from 0 to 720 hours; R^2^-value is the value for regression co-efficient.

## Conclusion

Nanoparticles calcium silicate powder doped with copper ions was prepared using wet precipitation method. CuO concentration influences the morphological properties, drug delivering ability, physicochemical properties and cytotoxicity. The results confirmed the dependence of the studied properties on the CuO content. In addition, very minor cytotoxicity effect was observed on normal cells compared to the cytotoxicity effect on the cancer cells. CuO-doped materials showed significant anticancer and it is recommended to be used in the treatment of cancer. And so, the future triumph of copper coordination compounds in the clinic demands close associations between biomedical scientists, clinicians and chemists.

## Ethical Issues

Not applicable.

## Conflict of Interest

The authors declare that there is no conflict of interest with any organization, authors and reviewers.

## Acknowledgments

This research work was not funded by any national or international organizations

## References

[1] Wu C, Fan W, Zhu Y, Gelinsky M, Chang J, Cuniberti G (2011). Multifunctional magnetic mesoporous bioactive glass scaffolds with a hierarchical pore structure. Acta Biomater.

[2] Lu H, Zhang T, Wang XP, Fang QF (2009). Electrospun submicron bioactive glass fibers for bone tissue scaffold. J Mater Sci Mater Med.

[3] Pretto M, Costa AL, Landi E, Tampieri A, Galassi C (2003). Dispersing behaviour of hydroxyapatite powders produced by wet-chemical synthesis. J Am Ceram Soc.

[4] Jamuna-Thevi K, Daud NM, Abdul Kadir MR, Hermawan H (2014). The influence of new wet synthesis route on the morphology, crystallinity and thermal stability of multiple ions doped nanoapatite. Ceram Int.

[5] Imrie FE, Skakle JMS, Gibson IR (2013). Preparation of Copper-Doped Hydroxyapatite with Varying x in the Composition Ca_10 _(PO_4_)_6_Cu_x_O_y_H_z_. Bioceram Dev Appl S1.

[6] Dornbusch K (1976). The detection of doxycycline activity in human bone. Scand J Infect Dis Suppl.

[7] Hamzawy EMA, Kenawy SH, Abd El Aty AA, El-Bassyouni GT (2018). Characterization of wollastonite-copper nanoparticles synthesized by a wet method. Interceram Int Ceram Rev.

[8] Gnarpe H, Dornbusch K, Hägg O (1976). Doxycycline concentration levels in bone, soft tissue and serum after intravenous infusion of doxycycline: A clinical study. Scand J Infect Dis Suppl.

[9] Spellberg B, Lipsky BA (2012). Systemic antibiotic therapy for chronic osteomyelitis in adults. Clin Infect Dis.

[10] Hyun S, Kim SG, Kweon HY, Jo YY, Lee KG, Kang TY (2014). Comparison of Different Concentrations of Tetracycline-loaded Silk Fibroin Membranes on the Guided Bone Regeneration in the Rat Calvarial Defect Model. Tissue Eng Regen Med.

[11] Oh SH, Nam BR, Lee IS, Lee JH (2016). Prolonged anti-bacterial activity of ion-complexed doxycycline for the treatment of osteomyelitis. Eur J Pharm Biopharm.

[12] Thabrew MI, Hughes RD, McFarlane IG (1997). Screening of hepatoprotective plant components using a HepG2 cell cytotoxicity assay. J Pharm Pharmacol.

[13] El-Menshawi BS, Fayad W, Mahmoud K, El-Hallouty SM, El-Manawaty M, Olofsson MH (2010). Screening of natural products for therapeutic activity against solid tumors. Indian J Exp Biol.

[14] Bassyouni FA, Abu-Baker SM, Mahmoud K, Moharam M, EL-Nakkady S, Abdel Rehim M (2014). Synthesis and biological evaluation of some new triazolo[1,5-a]quinoline derivatives as anticancer and antimicrobial agents. RSC Adv.

[15] Phaechamud T, Chanyaboonsub N, Setthajindalert O (2016). Doxycycline hyclate-loaded bleached shellac in situ forming microparticle for intraperiodontal pocket local delivery. Eur J Pharm Sci.

[16] Mabrouk M, Choonara YE, Kumar P, du Toit LC, Pillay V (2016). The Influence of Lyophilized EmuGel Silica Microspheres on the Physicomechanical Properties, In Vitro Bioactivity and Biodegradation of a Novel Ciprofloxacin-Loaded PCL/PAA Scaffold. Polymers.

[17] Cao L, Weng W, Chen X, Zhang J, Zhou Q, Cui J (2017). Effects of mesoporous calcium magnesium silicate on setting time, compressive strength, apatite formation, degradability and cell behaviour to magnesium phosphate based bone cements. RSC Adv.

[18] Costa P, Sousa Lobo JM (2001). Modeling and comparison of dissolution profiles. Eur J Pharm Sci.

[19] Ismail H, Shamsudin R, Azmi M, Abdul Hamid MA, Awang R (2016). Characteristics of β-wollastonite derived from rice straw ash and limestone. J Australia Ceram Soc.

[20] Puntharod R, Sankram C, Chantaramee N, Pookmanee P, Haller KJ (2013). Synthesis and characterization of wollastonite from egg shell and diatomite by the hydrothermal method. J Ceram Process Res.

[21] Gražėnaitė E, Kiuberis J, Beganskienė A, Senvaitienė J, Kareiva A (2014). XRD and FTIR characterisation of historical green pigments and their lead-based glazes. Chemija.

[22] Yu P, Kirkpatrick RJ, Poe B, McMillan PF, Cong X (1999). Structure of Calcium Silicate Hydrate (C-S-H): Near-,Mid- and Far-Infrared Spectroscopy. J Am Ceram Soc.

[23] Mabrouk M, Choonara YE, Marimuthu T, Kumar P, du Toit LC, van Vuuren S (2016). Ca_3_(PO_4_)_2_ precipitated layering of an in situ hybridized PVA/Ca_2_O_4_Si nanofibrous antibacterial wound dressing. Int J Pharm.

[24] Zhang H, Ye XJ, Li JS (2009). Preparation and biocompatibility evaluation of apatite/wollastonite-derived porous bioactive glass ceramic scaffolds. Biomed Mater.

[25] Tsigkou O, Labbaf S, Stevens MM, Porter AE, Jones JR (2014). Monodispersed bioactive glass submicron particles and their effect on bone marrow and adipose tissue-derived stem cells. Adv Healthc Mater.

[26] Kalaivani S, Singh RK, Ganesan V, Kannan S (2014). Effect of copper (Cu^2+^) inclusion on the bioactivity and antibacterial behaviour of calcium silicate coatings on titanium metal. J Mater Chem B.

[27] Jose GP, Santra S, Mandal SK, Sengupta TK (2011). Singlet oxygen mediated DNA degradation by copper nanoparticles: potential towards cytotoxic effect on cancer cells. J Nanobiotechnology.

[28] Dasari S, Tchounwou PB (2014). Cisplatin in cancer therapy: molecular mechanisms of action. Eur J Pharmacol.

[29] Denoyer D, Masaldan S, La Fontaine S, Cater MA (2015). Targeting copper in cancer therapy: ‘Copper That Cancer’. Metallomics.

[30] Jany T, Moreth A, Gruschka C, Sischka A, Spiering A, Dieding M (2015). Rational design of a cytotoxic dinuclear cu2 complex that binds by molecular recognition at two neighboring phosphates of the DNA backbone. Inorg Chem.

